# Endocrine-Disrupting Effects of Transplacental and Translactational Exposure to Tembotrione on Hormone Status in Wistar Rat Offspring at Different Developmental Stages: A Pilot Study

**DOI:** 10.3390/toxics12080533

**Published:** 2024-07-24

**Authors:** Anja Katić, Irena Brčić Karačonji, Vedran Micek, Davor Želježić

**Affiliations:** 1 Division of Toxicology, Institute for Medical Research and Occupational Health, Ksaverska cesta 2, 10000 Zagreb, Croatia; ibrcic@imi.hr (I.B.K.); dzeljezi@imi.hr (D.Ž.); 2 Faculty of Health Studies, University of Rijeka, Viktora Cara Emina 5, 51000 Rijeka, Croatia; 3 Animal Breeding Unit, Institute for Medical Research and Occupational Health, Ksaverska cesta 2, 10000 Zagreb, Croatia; vmicek@imi.hr

**Keywords:** triketone herbicide, pregnancy, lactation, 17β-estradiol, testosterone, rats

## Abstract

Green agronomy promotes the implementation of natural and naturally derived substances in crop protection. In the present study, we evaluated the endocrine-disrupting potential of the allelopathic herbicide tembotrione in Wistar rats by studying the hormone status of offspring from the treated dams. Three doses of tembotrione (0.0004, 0.0007, and 4.0 mg/kg b.w./day) have been administered to dams during gestation and/or lactation. In the serum of newborn, weaning, and pubertal female and male offspring, 17β-estradiol and testosterone were determined using enzyme-linked immunosorbent assay. A decrease in 17β-estradiol and testosterone was observed in female and male weaning and pubertal offspring exposed to all doses of tembotrione during gestation and lactation. In weaning offspring exposed only during lactation, 17β-estradiol dropped significantly after exposure to the two lower doses and testosterone after exposure to the lowest dose of tembotrione. The greatest effect was observed at the lowest dose of tembotrione. In newborns, we observed increased 17β-estradiol after exposure to two lower doses of tembotrione and significantly increased testosterone after exposure to the lowest dose. The highest dose of tembotrione decreased 17β-estradiol significantly in newborn females. The obtained results suggest that tembotrione might be considered a pro-estrogenic or estrogen agonistic compound under the exposure conditions applied in this investigation.

## 1. Introduction

Allelopathic herbicides are substances synthesized by plants that impair the growth of plants of other genera but stimulate their own. Tembotrione is a chemical allelopathic triketone herbicide [1], approved by the European Commission in 2006 as an active substance authorized for incorporation in plant protection products [2]. It is used post-emergently to control broadleaf and grassy weeds. In the plant, it inhibits 4-hydroxyphenylpyruvate dioxygenase (HPPD), the crucial enzyme for the formation of homogentisic acid and for the synthesis of plastoquinone and tocopherols that are indispensable in the protection of chloroplast in plants due to oxidative damage protection that enables photosynthesis in thylakoid membranes [3,4]. As far as the authors are aware, there is no published data regarding the toxicokinetics or toxicodynamics of tembotrione in humans. In rats, it is rapidly absorbed and extensively metabolized, mostly to 4-hydroxy- and 5-hydroxy-tembotrione. More than 96% of the paternal molecule is excreted via urine and feces within the first 24 h [5].

To ease the classification of chemicals as endocrine-disrupting chemicals (EDCs), the European Chemicals Agency (ECHA) and the European Food Safety Authority (EFSA) jointly issued the guidance document [6] in accordance with the endocrine disruption (ED) criteria laid down in Commission Delegated Regulation (EU) No 2017/2100 [7] and Commission Regulation (EU) No 2018/605 [8] for biocidal products (BP) and plant protection products (PPP), respectively. EDCs can act by interfering with hormone binding to the corresponding receptor and on enzymes of steroidogenic and metabolic pathways. Apart from disrupting hormone receptor activation resulting in genomic response, EDCs can also induce a rapid non-genomic response by binding plasma membrane receptors and through second messenger-triggered signal cascades [9,10]. Epigenetic changes present the possible mechanisms linking exposure to EDCs and adverse outcomes in later life and in future generations [11].

Many pesticides have been recognized as potent ECDs, causing different health effects in humans and other species that can even be multi- and transgenerational [12,13,14,15,16]. Numerous in vivo and in vitro studies have demonstrated that EDCs, including pesticides, interfere with sex steroid hormone biosynthesis and metabolism [17,18,19]. Triketone herbicides, to which tembotrione belongs, are suspected as possible EDC to many organisms [20,21,22,23,24].

In this pilot study, the serum levels of two sex steroid hormones, estrogen 17β-estradiol and androgen testosterone, in female and male offspring of dams treated with tembotrione, served as the endpoints of transplacental and/or translactational ED activity of tembotrione. The levels of hormones were evaluated at different stages of offspring development and maturation.

The placenta, as a temporary organ that plays a key role in fetal growth and development, presents an important barrier to fetal protection during pregnancy, but it is not entirely impermeable. Many studies have confirmed that EDCs, including pesticides, pass over the placental barrier, enter the fetal/embryonic blood, and induce adverse effects in the fetus/embryo [25,26,27,28].

Furthermore, there are many studies showing that persistent organic pollutants, such as pesticides, are transferred during lactation through breast milk from the mother to her sucklings [29,30,31,32,33,34].

The most sensitive subpopulation to the adverse effects of pesticides are children who can be exposed to agrochemicals even from the first day of fetal development through the placenta and later through breast milk [35]. It is well-known today that early exposure during sensitive periods of development can present a risk for diseases in later life, known as the developmental origins of health and disease (DOHaD) concept [36].

Therefore, we proposed the hypothesis that transplacental and translactational tembotrione exposure of female and male rat offspring could impair the levels of the two most important steroid sex hormones, 17β-estradiol and testosterone. To test this hypothesis, we focused on a rat experimental model and used animals at three different stages of development: newborn, weaning, and pubertal rat offspring. Dams were treated with selected tembotrione doses from conception to weaning. We focused on low tembotrione doses relevant for real human exposure according to the EFSA [37].

## 2. Materials and Methods

### 2.1. Chemicals and Reagents

Tembotrione 99.9% (CAS-No. 335104-84-2), as the analytical standard PESTANAL^®^, and the positive control, ethinyl estradiol (EE) ≥ 98.0% (a/a) (CAS-No. 57-63-6), were purchased from Sigma-Aldrich Laborchemikalien GmbH, Seelze, Germany. To obtain the doses used in the treatments, tembotrione and EE were dissolved and diluted in deionized water purified with a Milli-Q water purification system (Millipore, Bedford, MA, USA).

### 2.2. Animals

Three-month-old, sexually mature, female and male Wistar rats (*Rattus norvegicus* sp.) were grown in the breeding colony at the Animal Breeding Unit of the Institute for Medical Research and Occupational Health, Zagreb, Croatia. All animals were under constant veterinary health care. The study was performed on a total of 25 female rats, with an initial average body weight of 240 g.

During the experiment and until the moment of sacrifice, the animals were housed in accordance with the standard operating procedures that refer to the treatment of animals in the experiment in the Animal Breeding Unit of the Institute for Medical Research and Occupational Health. Animals were kept in standard clear polycarbonate cages (Ehret, Tulln, Austria) with *ad libitum* access to standard Good Laboratory Practice (GLP)-certified food (Complete feed for mice and rats 4RF21, Mucedola, Settimo Milanese, Italy) and tap water under pathogen-free conditions in a steady-state microenvironment at room temperature, 20–22 °C, with a 12-h light/dark cycle and humidity of 40–60%. The husbandry was kept sterile and detached from either of the nearby rooms. The rats were kept on wood litter in the form of wood shavings. During the entire experiment, cage hygiene was carried out regularly. The physiological and ethological requirements of the animal/group of animals were considered when placing them in cages, and an optimal living space was provided while taking care of their well-being and respecting the regulations and guidelines from modern practice in working with laboratory animals [38]. The living space of the experimental animals was enriched in such a way that the animals could show the full range of their usual behavior without risking the outcome of the research (cardboard houses, tunnels, wooden blocks for nibbling). Nesting material (paper tissues) was added to pregnant rats for nesting. Cardboard tubes were not an option for entertaining pregnant rats, as they gained volume rapidly and would get stuck. The study was approved by the Institutional Animal Care and Use Committee and the Croatian Ministry of Agriculture. It was carried out in compliance with international standards and national legislation to protect animal welfare.

### 2.3. Experimental Design

After identifying rats with a regular 4-day estrous cycle, on the day of pro-estrous regular cycling, female rats were mated with unexposed sexually mature males overnight in a 2:1 ratio. The following morning, pregnancy was ascertained by the presence of sperm in a vaginal lavage or copulation plug in the vagina; that day was designated as Gestation Day 1 (GD1). Pregnant rats were assigned into control and treatment groups comprising five animals with similar average body weights.

To determine the minimum number of offspring needed for analysis to enable us to monitor possible endocrine-disrupting effects with the animals’ welfare in mind, a power analysis was performed. We considered the number of treatment groups, negative and positive controls, known reference values of measured parameters, and the expected extent of their change by applying standard parameters for the level of significance (α = 0.05) and minimum power (β = 0.80). For analysis, we used the G*Power software, version 3.1., according to Faul et al. [39]. The power analysis showed that five pups per treatment group were sufficient to detect monitored adverse effects.

To achieve the largest possible uniformity, after birth, litters from the dams that brought more than five male and five female pups were culled randomly. However, attention was paid to pups of similar weight and activity. When the number of live offspring in the litter was less than five of each sex, the litter size was adjusted to ten by including another brood offspring from the same treatment or control group. An additional brood was chosen on the criteria of similar weight and activity of the pups.

Three different doses of tembotrione, relevant for both residential and occupational human exposure, were chosen for assessing transplacental and/or translactational effects on the level of sexual hormones in the serum of F1 offspring. They were within the range of low doses as defined by EFSA [37]; the first one corresponds to the value determined as Acceptable Daily Intake (ADI)—0.0004 mg tembotrione/kg b.w./day—and the second one is Accepted Operated Exposure Level (AOEL)—0.0007 mg tembotrione/kg b.w./day. For the third, the highest dose was 1/500 LD_50_ (median lethal dose), i.e., 4.0 mg tembotrione/kg b.w./day [37]. The importance of recording data on the toxicity of low doses is stressed in the Directive on the Sustainable Use of Pesticides by the EU Parliament and Council [40]. Negative controls received water and appropriate positive controls received orally active synthetic estrogen ethinylestradiol (EE) in concentrations of 50 µg/kg b.w./day [41,42]. All groups of animals were treated orally by gavage, with 1 mL of appropriate solution. During gestation, treatment with tembotrione and water lasted during the whole gestation period, while treatment with EE started on tGD7 and continued through the day of birth (±GD21). All animals were handled in the same manner. The experimental schedule is listed in Table 1.

During the experiment, body weights were regularly monitored two times weekly during the first two weeks of gestation and during lactation, and every day during the third week of gestation, and the doses of tembotrione and EE were adjusted accordingly. Survival and clinical signs of intoxication, as well as behavioral changes, were monitored on a daily basis. During gestation, attention was paid to possible bleeding and miscarriages.

### 2.4. Sample Collection

Transplacentally exposed newborn rats were sacrificed by decapitation within 24 h after birth. The blood samples from five newborns of each gender per group were collected in heparinized vacutainers (Becton Dickinson, Franklin Lakes, NJ, USA) for hormone analyses in serum.

At weaning day (PND21), offspring were sacrificed to assess the joint effect of transplacental and translactational exposure, as well as exposure through breast milk itself by using an anesthetic cocktail (Narketan, Vetoquinol UK Ltd., Towcester, UK, 80 mg/kg b.w.; Xylapan, Vetoquinol UK Ltd., 12 mg/kg b.w., i.p.). Blood from five female and five male offspring per group was collected by dissection of the carotid artery under general anesthesia for serum hormone level analyses.

To evaluate the possible endocrine-disrupting effect of tembotrione on the level of 17β-estradiol in pubertal female and testosterone in pubertal male offspring, as the result of continuous transplacental and translactational exposure until PND21, hormone levels were assessed at the beginning of puberty. External signs of sexual maturation (the onset of puberty) were monitored on a daily basis from the fifth week of the offspring’s life. In male offspring, preputial separation was monitored, and in the female offspring, the vaginal opening and estrous cycle were monitored [43]. After confirmation of puberty onset, five offspring of each gender from all experimental groups were sacrificed as described previously. The blood samples for hormone analyses in serum were sampled from the carotid artery.

### 2.5. Analysis of Steroid Hormones in Serum

The collected blood samples were centrifuged at 3000 rpm, i.e., 976 g, 15 min, and at 4 °C, twice, and the serum was separated and stored at −20 °C until analysis.

The 17β-estradiol and testosterone serum levels were measured by enzyme-linked immunosorbent assay (ELISA) using the Estradiol rat ELISA Kit (DEV9999) (Biomatik, Kitchener, ON, Canada) and Testosterone rat/mouse ELISA Kit (DEV9911) from Demeditec Diagnostics GmbH (Kiel, Germany), according to the standard protocol supplied by the kit manufacturer. The absorbance of each well was read at 450 nm using a Personal Lab instrument (IASON, Graz, Austria).

### 2.6. Statistical Analysis

To test the normality of distribution regarding the parameter of concern, we used the Shapiro-Wilk W test. Before data analysis, to obtain normal distribution, data were log_10_ transformed. Multiple comparisons between groups were performed by one-way analysis of variance (ANOVA) followed by a *post-hoc* Tukey HSD test. The results were expressed as mean ± standard error of the mean (SEM).

The statistical analysis was performed by Dell™ Statistica™-licensed statistical software package, version 14.0.0.15 (TIBCO Software Inc., Palo Alto, CA, USA), and Prism 9 software (GraphPad Software, Boston, MA, USA). The difference between groups was considered significant if *p* < 0.05. For power analysis, we used the software G*Power, version 3.1., according to Faul et al. [39] by applying standard parameters for the level of significance (α = 0.05) and minimum power (β = 0.80).

## 3. Results

### 3.1. 17β-Estradiol Concentrations in the Serum

**Figure 1:**
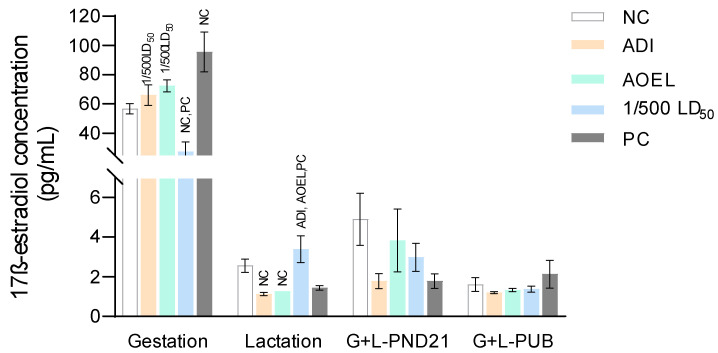
17β-estradiol concentrations (pg/mL) in the serum of female rat offspring after exposure to tembotrione and to the respective controls through dams during gestation (G), lactation (L), gestation and lactation at weaning (on PND 21), and gestation and lactation at the onset of puberty (PUB). Results are presented as mean ± standard error of the mean (SEM). Comparisons between samples were done using ANOVA and a *post-hoc* Tukey HSD test at *p* < 0.05. This was significantly different compared to the: NC–negative control; PC–positive control; ADI–Acceptable Daily Intake (0.0004 mg/kg/day); AOEL–Accepted Operated Exposure Level (0.0007 mg/kg/day); and 1/500 LD_50_ (median lethal dose) (4.0 mg/kg/day).

The concentrations of 17β-estradiol in the serum of female offspring exposed to tembotrione and the respective controls through dams during gestation and/or lactation are presented in Figure 1.

Considering the possible transplacental or translactational effect of tembotrione, a certain level of fluctuations and inconsistencies in the results may be observed in all offspring groups. However, the highest dose of tembotrione (4.0 mg/kg b.w./day) reduced 17β-estradiol production in all of the female offspring groups, except in the weaning pups of dams treated only during the lactation period. This decrease in 17β-estradiol concentration was significant compared to the negative controls in the newborn offspring exposed during gestation. In the female offspring of dams treated with two lower doses of tembotrione (0.0004 and 0.0007 mg/kg b.w./day) during gestation, a dose-related increase was recorded in the 17β-estradiol concentration, but it was not significant compared to the negative control group. However, it was perceived that both lower doses of tembotrione decreased the 17β-estradiol concentration in the weaning female offspring of dams treated only during lactation and during gestation and lactation, and in the pubertal female offspring of dams treated in gestation and lactation. The decrease was significant compared to the negative control group in the weaning offspring of dams treated during lactation only. This effect of lowering 17β-estradiol concentration was greater at the lowest dose of tembotrione (0.0004 mg/kg b.w./day) in all groups of female offspring. The levels of 17β-estradiol in the positive control group followed the change in the direction of the levels observed in the female offspring of dams treated with two lower doses of tembotrione in all groups except in the pubertal female offspring exposed through dams during gestation and lactation.

No effects of tembotrione and EE exposure were observed on pubertal onset in female offspring.

### 3.2. Testosterone Concentrations in the Serum

**Figure 2:**
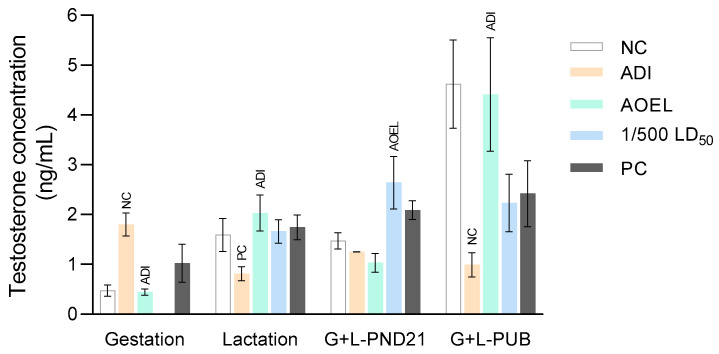
Testosterone concentrations (ng/mL) in the serum of male rat offspring after exposure to tembotrione and to the respective controls through dams during gestation (G), lactation (L), gestation and lactation at weaning (on PND 21), and gestation and lactation at the onset of puberty (PUB). Results are presented as mean ± standard error of the mean (SEM). Comparisons between samples were done using ANOVA and a *post-hoc* Tukey HSD test at *p* < 0.05. This was significantly different compared to the: NC–negative control; PC–positive control; ADI–Acceptable Daily Intake (0.0004 mg/kg/day); and AOEL–Accepted Operated Exposure Level (0.0007 mg/kg/day).

Changes in testosterone concentrations measured in the serum of male offspring exposed to tembotrione and the respective controls through dams during gestation and/or lactation are presented in Figure 2.

As the tembotrione doses were administered to dams by the same scheme as for the female offspring, similarly to the 17β-estradiol concentrations in the serum of female offspring, there were some discrepancies and fluctuations in the testosterone concentrations in all of the male offspring groups. Much like the effects on the 17β-estradiol concentrations, the lowest dose of tembotrione (0.0004 mg/kg b.w./day) decreased the concentrations of testosterone in the weaning male offspring treated only during lactation, two lower doses of tembotrione (0.0004 and 0.0007 mg/kg b.w./day) decreased testosterone concentrations in the weaning male offspring treated during gestation and lactation, and exposure to all three doses of tembotrione decreased testosterone concentrations in pubertal males. Only the lowest dose of tembotrione (0.0004 mg/kg b.w./day) had a significant impact on testosterone decrease in the pubertal male offspring of dams treated in gestation and lactation compared to negative controls. Also, in male offspring of dams treated during gestation with tembotrione at the same dose, testosterone concentrations significantly increased compared to negative controls, while the dose of 0.0007 mg/kg b.w./day slightly decreased testosterone concentrations. The highest dose of tembotrione (4.0 mg/kg b.w./day) reduced testosterone concentrations only in pubertal male offspring, while in weaning male offspring treated during lactation only, and during gestation and lactation testosterone, concentrations were increased, but these changes were not significant compared to negative controls. The testosterone concentrations in the positive control group followed the change in the direction of the values observed in the highest dose of tembotrione in all groups. Due to the small amount of the newborns’ male rat serum, data on the testosterone concentration are missing in the group exposed to the highest dose of tembotrione (4.0 mg/kg b.w./day).

There was no effect on pubertal onset in male offspring after tembotrione and EE exposure.

## 4. Discussion

The results obtained in the present study confirmed the proposed hypothesis and indicated that indirect exposure to the triketone herbicide tembotrione through the placenta and/or maternal milk may lead to ED of sex hormones of F1 female and male offspring in different stages of development until puberty.

We decided to follow the proposed study design and focused on offspring because the pre- and post-natal periods are characterized by embryogenesis as well as organ development and maturation, especially of the reproductive and neural systems. This makes these periods the most critical and delicate for the adverse activity of toxic agents, including herbicides, to which offspring may not be exposed to directly but can be through the placenta or maternal milk [33,44]. Besides, triketone herbicides are considered possible EDCs [24]. The endocrine-disrupting effects of the triketone herbicide atrazine, which has been banned in the European Union since 2004, including disruption of steroid hormones, are well-known [13], while there are no data on the endocrine-disrupting effects of tembotrione and particularly not on the most sensitive populations.

Until today, there have been many investigations in humans and laboratory animals that showed the transplacental and translactational endocrine-disrupting effects of pesticides with adverse consequences on the health of the offspring [28,33]. There is evidence that tembotrione may be transferred by lactation from lactating cows treated twice daily for seven consecutive days at 0.037 mg tembotrione/kg b.w. per day and 0.41 mg tembotrione/kg b.w. per day to suckling calves. The same results were found for its metabolite, M5 (dihydroxy-tembotrione). However, low recovery of the administered dose was detected in milk and there was no dose level correlation [45].

Along with the other xenobiotics, the applied tembotrione does not reach the fetus/embryo as a parental molecule only, but rather also possesses the potential to be transformed into toxic metabolites. It has to pass the maternal liver and placenta, two major metabolically active organs, while the fetal liver has a lower concentration of detoxifying enzymes due to a primitive metabolism in comparison to an adult [46,47].

Considering the mechanism of action (MoA) of EE, which has been used as a positive control in the present study and in general in studies of ED activities of different agents, it acts as an estrogen agonist. More precisely, it binds to estrogen receptors with multiple-times higher affinity than 17β-estradiol itself [48,49]. By binding the estrogen receptors, a perception is generated that the level of endogenous estradiol is too high [50]. Thus, exposure to EE for 21 or 42 consecutive days in our study should downregulate the synthesis of endogenous 17β-estradiol by the mechanism of negative feedback regulation through the hypothalamic-pituitary-ovary axis. As a result, we observed lower serum levels of 17β-estradiol in weaning female offspring exposed to EE during lactation only and during gestation and lactation compared to negative controls, but not significantly. In groups of female offspring exposed to two lower doses of tembotrione (0.0004 and 0.0007 mg/kg b.w./day) only during lactation 17β-estradiol levels were significantly reduced, and the same effect, though not significant, was observed in weaning and pubertal female offspring exposed to tembotrione during gestation and lactation. Also, the highest applied dose of tembotrione (4.0 mg/kg b.w./day) resulted in significantly decreased 17β-estradiol levels in female offspring exposed during the gestation period as well as decreased 17β-estradiol levels in weaning and pubertal female offspring exposed to tembotrione during gestation and lactation. Investigation on human placental JEG-3 cells has shown that estradiol drops under the influence of pro-estrogenic substances are a result of an increase in CYP1A1 protein levels and inhibition of CYP19A1 protein levels [51]. The exact mechanism is not known yet, but both proteins are involved in estradiol synthesis and catabolism. The greatest effect of 17β-estradiol reduction observed in the weaning and pubertal rats at the lowest dose of tembotrione could be explained by one of the most important characteristics of EDCs, i.e., low-dose effect. This characteristic of EDCs is connected with non-monotonic dose-response curves (NMDRCs), which are U-shaped or inverted U-shaped curves, and that makes it impossible to predict the effect of low doses by the effects at high doses, as it is common for other toxic chemicals. Risk assessments of EDCs are particularly challenging due to this reason: it is hard to determine a safe dose or a threshold [41]. According to Beausoleil et al. [52], although the mechanisms for NMDRCs are still unknown, they should not be neglected.

Estradiol plays a crucial role in prenatal and early neonatal neural and brain development, in respiratory and metabolic control in newborn rats, as well as in the development of secondary sexual characteristics and in the control of reproductive functions in women [53,54,55]. By regulating the metabolic effects of the growth hormone that controls growth and development, estradiol affects sexual dimorphism, and in puberty, it controls the successful completion of body composition for each gender [56]. Since estrogen acts through specific receptors, estrogen receptors (ERs), which are transcriptional factors expressed on the membrane surface of many different cells, it is obvious that estradiol is important for the proper development of different tissues and organs.

In rat fetuses and newborns, estradiol originates from maternal circulation and from themselves and is synthesized preferentially in the brain over gonads in female rats [57,58]. It is well-known that estradiol, the activity of aromatase cytochrome P450 (CYP19), which is the enzyme that converts testosterone to estradiol, as well as estrogen receptors, is at a very high level in the prenatal brain and during the few first days after birth [57,58]. Since rat ovarian follicle development starts after birth [59,60], evidence suggests that rodent ovaries start with significant estradiol secretion after PND7 [61]. This period of postnatal ovarian activity when estradiol and gonadotropin surge happen is called “mini-puberty” and lasts from the 10th to 20th PND in rats [62]. At the beginning of puberty, which occurs around the 35th to 45th PND in rats, estradiol concentrations increase preceding the first events of puberty, vaginal opening, and first ovulation [43]. We observed that concentrations of 17β-estradiol after exposure during gestation were more than one order of magnitude higher than in the weaning and pubertal female offspring in all experimental groups, which could be explained by maternal origin due to high estradiol synthesis during the gestation period in rats, with its peak on the 19th day of gestation. Around the 20th to 22nd day of rat gestation, and at the beginning of lactation, estradiol levels decline due to elevated prolactin levels and remain mostly stable during the rest of lactation [63]. Also, our results have shown that serum levels of 17β-estradiol were higher in weaning offspring on PND21, in the so-called “mini-puberty” period, compared to pubertal offspring, both exposed during gestation and lactation, in all experimental groups except in the positive control.

Besides lowering the level of 17β-estradiol in female offspring, in male offspring, indirect exposure to tembotrione and EE through placenta and/or breast milk decreased testosterone levels in the serum at some sampling points. In pubertal male offspring, tembotrione and EE exposure led to deprivation of testosterone concentrations, with a significant effect at a dose of 0.0004 mg tembotrione/kg b.w./day. The same effect of EE on decreased serum testosterone concentrations in pubertal male pups of F1 and F2 generation, as the result of exposure through feed containing 10 and 50 ppb EE before and during gestation and lactation until the day of sacrificing on PND50, was reported in the NIH’s toxicology study [64]. Decreased levels of testosterone were also observed in newborn and weaning male offspring after exposure to two lower doses of tembotrione during gestation and/or lactation.

In this study, tembotrione did not affect testosterone serum levels following the monotonic dose-response curve, which is the same as was stated earlier for 17β-estradiol levels. As proven, low doses of a toxic substance do not have to follow a linear or exponential curve to be considered endocrine toxic. Moreover, they may follow NMDRCs of different shapes/curves, as was shown in our study for both steroid hormones. Nevertheless, the non-monotonic response of chemicals has been observed at least ten years ago [65].

Testosterone levels may be regulated by the hypothalamic–pituitary–testicular axis. Sub-chronic exposure to pro-estrogen may inhibit the release of gonadotropin and luteinizing hormone (LH), which could downregulate testosterone synthesis [66]. There is evidence that suppression of Leydig cell function in terms of steroidogenesis, due to estrogen exposure, may be mediated by ERα-dependent signaling. This MoA includes attenuation of Nur77, a transcription factor that regulates the expression of several steroidogenic genes [67]. The drop in testosterone levels under exposure to exogenous estrogens may also be the consequence of downregulation of androgen receptor expression [68]. As postulated by Williams [68], to disturb male reproductive organs, decreased levels of testosterone alone are not sufficient; they should also be accompanied by a simultaneous increase in estrogen action. Both of these criteria were met in the pubertal male offspring exposed to both EE and the highest dose of tembotrione in our investigation.

Unlike the female gonads, which are inactive during fetal life, testosterone production in the testes starts around the 15th day of gestation and reaches its peak around the 17th day of gestation, independently of gonadotropin LH regulation [69]. Investigations in rats have shown that the critical period of androgen-dependent masculinization occurs between embryonic day 15.5 to 17.5, which follows the onset of testosterone production in the testes and is called the “masculinization programming window” [70]. During the masculinization period, the differentiation of the internal and external genitalia occurs, while masculinization of the brain occurs perinatally in rats [70]. After that, testosterone levels fall and six hours after birth a testosterone surge occurs, with its peak two hours later, as is also the case with gonadotropin LH [71]. The “mini-puberty” period is considered to be an important stage for sexual development and a critical window of programming with long-life implications [72]. Testes continue testosterone biosynthesis at puberty, stimulated by the hypothalamic-pituitary hormones, to initiate the development of secondary male sex characteristics, sexual function, and spermatogenesis [73]. Low levels of serum testosterone in newborn rats in our investigation were an order of magnitude lower in the negative control and tembotrione groups (0.0007 mg/kg b.w./day) and about two times lower in the EE group in comparison with pubertal rats, which could be explained by the fact that we measured hormone levels in serum collected within 24 h after birth when the testosterone surge during “mini-puberty” period had already passed.

Concerning the observed adverse effects of EE in this investigation, there may exist the possibility that the negative effects of xenoestrogen on estradiol and testosterone levels would be even more pronounced in humans over rats because, unlike in humans, the rat uterus is capable of metabolizing a certain amount of EE to the less potent estrogen estrone due to the presence of the UDP-glucuronosyl transferase enzyme [74].

Estrogen and androgen hormones are the most important factors in the normal growth and reproductive function of the organism. EDCs can be estrogenic or androgenic disruptors, or both, and can cause developmental and reproductive defects in males and females. Many pesticides with ED properties may act as xenoestrogens or antiandrogens. As a consequence, frequent feminization of males of different animal species takes place [75,76]. The issue of male feminization represents a continuously growing problem in many countries because it may result in infertility [77,78,79,80,81]. Besides effects on male reproduction, exposure to pesticides can affect female reproduction by multiple mechanisms and correlate with female infertility [77,82,83].

As already mentioned, the in-utero and breast-feeding periods are especially sensitive periods in life when sexual differentiation and gonadal development, as well as the production of steroid sexual hormones, occur. Exposure to anti-androgenic EDCs during these periods may result in many alterations such as decreased anogenital distance (AGD), increase in retention of areolae and/or nipples, epididymal agenesis, decrease in sex accessory gland weights, cryptorchidism, hypospadias and reduced fertility in male offspring, with little effect in female offspring [84]. Since the masculinization process is completely hormone-dependent, exposure to any estrogenic or anti-androgenic EDC can disrupt this process via many pathways [85]. Estrogenic EDCs may also have different adverse effects on female reproductive health since both breast and uterine tissues are very sensitive to estrogens and, accordingly, may play an important role in the early onset of puberty and delayed menopause [86]. Various mechanisms by which environmental compounds may affect these hormone-dependent processes and cause reproductive diseases in both sexes through androgen or estrogen pathways, or both, on the genetic and epigenetic level, have been investigated during the past several decades [87]. The diversity of pesticides’, including herbicides’, ED mechanisms of actions and adverse effects, including reproductive effects, are summarized in Warner et al. [88].

The effect of some compounds on hormone levels, as we investigated in our study, presents one of the key events (KE) in the adverse outcome pathway (AOP) cascade, and that may cause different adverse impacts on the reproductive systems of males and females. In our study, we did not investigate other KEs that can be connected with hormone disruption and lead to adverse outcomes in the AOP cascade for estrogenic or androgenic action, which could represent the limitation in its design.

Nevertheless, to the best of our knowledge, so far there have been no papers published regarding the endocrine or other reproductive toxic activity of tembotrione that would enable a comparison with our results. Among the available reports, there is one by Mikolić et al. [89], whose results showed that gestational and lactational exposure to tembotrione disturbed serum estradiol and testosterone levels in both female and male rats from birth until the onset of puberty.

## 5. Conclusions

The obtained results add new data regarding gestational and lactational tembotrione exposure on sex steroid hormone levels in newborn, weaning, and pubertal female and male rat offspring. Based on all of our observations, we may suggest that transplacental and translactational exposure to tembotrione provoked pro-estrogenic or estrogen agonistic effects in rat offspring until puberty. The importance of this study lies in the fact that we have shown for the first time that tembotrione exposure during sensitive periods of development, such as pregnancy and lactation, disrupts hormone levels in female and male rat offspring.

Disrupted sex steroid hormone levels caused by tembotrione exposure as the KE can be a starting point and part of the AOP for ED that will ultimately lead to adverse outcomes, together with other KEs that remain to be investigated. Also, this is the step forward to elucidation mode of action of tembotrione as an EDC on the reproductive system.

Our experimental approach has some limitations as well. We are aware that doses and analyses used in this study cannot completely clarify the possible endocrine-disrupting effects of tembotrione on the most sensitive population. Since this investigation was conducted as a small part of the larger study carried out in our institution within the framework of the project entitled “Organic pollutants in environment—markers and biomarkers of toxicity”, designed to test the effects of low doses of several insecticides and herbicides, testing additional doses and collecting biological material for additional analyses was not possible. However, the fact that selected doses have the potential to disrupt sex steroid hormone levels in offspring exposed through the placenta or maternal milk indicates their importance and calls for extensive research. Further studies should therefore consider analyses of other hormones such as thyroid hormones, gonadotropins LH, and FSH, as well as histopathological analyses of reproductive organs to help us elucidate the mechanisms of tembotrione ED effects on reproductive function. Besides, the determination of tembotrione levels in maternal and fetal tissue should help us to elucidate the transplacental and translactational transfer of tembotrione.

## Figures and Tables

**Table 1:** Experimental schedule.

Exposure	Duration of the Treatment	Time of Sampling
Transplacental	Entire gestation [(except for ethinylestradiol (EE)] Consecutive ± 21 days	Newborns 24 h after birth
Transplacental + translactational – until weaning at postnatal day (PND)21	Entire gestation (except for EE) + 21 day of lactation Consecutive ± 42 days	Offspring at PND21
Transplacental + translactational – until weaning at PND21	Entire gestation (except for EE) + 21 day of lactation Consecutive ± 42 days	Offspring at the onset of puberty
Translactational	21 days of lactation 21 consecutive days	Offspring at PND21

## Data Availability

The raw data supporting the conclusions of this article will be made available by the authors on request.
